# Rigorous Computational and Experimental Investigations on MDM2/MDMX-Targeted Linear and Macrocyclic Peptides

**DOI:** 10.3390/molecules24244586

**Published:** 2019-12-14

**Authors:** David J. Diller, Jon Swanson, Alexander S. Bayden, Chris J. Brown, Dawn Thean, David P. Lane, Anthony W. Partridge, Tomi K. Sawyer, Joseph Audie

**Affiliations:** 1 CMDBioscience, 5 Park Avenue, New Haven, CT 06511, USA; djrdiller@gmail.com (D.J.D.); jon@chemmodeling.com (J.S.); alexander@bayden.net (A.S.B.); 2 Venenum BioDesign, LLC, 8 Black Forest Road, Hamilton, NJ 08691, USA; 3 ChemModeling, LLC, Suite 101, 500 Huber Park Ct, Weldon Spring, MO 63304, USA; 4 Kleo Pharmaceuticals, 25 Science Park, Ste 235, New Haven, CT 06511, USA; 5 A*STAR, p53 Laboratory, Singapore 138648, Singapore; cjbrown@p53lab.a-star.edu.sg (C.J.B.); dawn.thean@gmail.com (D.T.); dplane@p53Lab.a-star.edu.sg (D.P.L.); 6 MSD International GmbH, Singapore 138665, Singapore; anthony_partridge@merck.com; 7 Merck Research Laboratories, 33 Avenue Louis Pasteur, Boston, MA 02115, USA; 8 College of Arts and Sciences, Department of Chemistry, Sacred Heart University, 5151 Park Avenue, Fairfield, CT 06825, USA

**Keywords:** peptide design, free energy calculation, d-amino acid scan, alanine scan

## Abstract

There is interest in peptide drug design, especially for targeting intracellular protein–protein interactions. Therefore, the experimental validation of a computational platform for enabling peptide drug design is of interest. Here, we describe our peptide drug design platform (CMDInventus) and demonstrate its use in modeling and predicting the structural and binding aspects of diverse peptides that interact with oncology targets MDM2/MDMX in comparison to both retrospective (pre-prediction) and prospective (post-prediction) data. In the retrospective study, CMDInventus modules (CMDpeptide, CMDboltzmann, CMDescore and CMDyscore) were used to accurately reproduce structural and binding data across multiple MDM2/MDMX data sets. In the prospective study, CMDescore, CMDyscore and CMDboltzmann were used to accurately predict binding affinities for an Ala-scan of the stapled α-helical peptide ATSP-7041. Remarkably, CMDboltzmann was used to accurately predict the results of a novel D-amino acid scan of ATSP-7041. Our investigations rigorously validate CMDInventus and support its utility for enabling peptide drug design.

## 1. Introduction

A renaissance in peptide drug discovery is underway, especially as it pertains to selectively targeting disease associated intracellular protein–protein interactions. Not surprisingly, many new chemical and biological technologies have been developed and are being used to enable the design and discovery of peptide research tools and drug candidates. Recent work points to an emerging role for information-based and physics-based computational methods to advance novel linear and macrocyclic peptide drug discovery. Here, we take an important step in that direction and describe the rigorous experimental validation of multiple computational methods and predictions in retrospective and prospective peptide conformational modeling and binding affinity studies on the well-known and clinically important p53-MDM2/MDMX systems. Our study is instructive in that it includes a broad range of MDM2/MDMX associated data—including data from linear peptides, D-peptides, stapled peptides, bicyclic peptides, and N–C cyclized peptides—and includes a prospective binding affinity study with computational predictions made using a range of methods prior to experimental testing. Importantly, the prospective binding affinity study strongly validates the use of our computational methods in guiding the design of α-helical peptides and shows how such computational methods, especially when combined with scientific ingenuity, can be used to model and propose novel peptide synthetic modifications.

For nearly three decades, MDM2 and MDMX have been aggressively pursued as oncology drug targets [1]. Briefly, p53 acts as a tumor suppressor by causing cell-cycle arrest and apoptosis in response to DNA damage. For this reason, it is often referred to as the guardian of the genome [2]. In many forms of cancer, p53 is inactivated either through mutation or through the over-expression of negative regulators, including MDM2 and MDMX. MDM2 and MDMX act to regulate p53 via interaction with its N-terminal transactivation domain, which corresponds approximately to residues 17–29. Thus, blocking the MDMX/MDM2/p53 interactions has been and remains a focal point in cancer drug discovery. Indeed, both biotech and pharma have serious interest in developing stapled α-helical peptide drugs to target the p53 binding sites of MDMX and MDM2. Specifically, Aileron Therapeutics is currently testing ALRN-6924, a dual MDM2/MDMX inhibitor in Phase I/II clinical trials [3].

Due to the intense interest over such a long period of time, a rich and complex data set has emerged around inhibitors of the MDM2/p53 and MDMX/p53 interactions. Collectively, this may represent the most extensive target data set available for novel peptide-based drug discovery. For example, there are at least 19 MDM2 and 9 MDMX crystal structures with co-crystallized peptides available in the Protein Data Bank (PDB). Further, many of the co-crystal structures have corresponding structure activity relationship (SAR) data available in the literature (see Table 1), with many having both MDM2 and MDMX SAR data.

The first major MDM2/MDMX-targeted SAR study used linear α-helical peptides comprised of all L-amino acids. Li and co-workers [4] published an extensive mutational analysis of MDM2 and MDMX binding to both p53 (residues 17–28 ETFSDLWKLLPE) and the more potent linear peptide PMI (TSFAEYWNLLSP). This dataset consists largely of an alanine scan of each peptide along with a handful of truncated peptides. Both peptides have been crystallized with MDM2 (p53 in 1ycr [5] and 4hfz [6], and PMI in 3eqs [7]). PMI has additionally been co-crystallized with MDMX (3eqy [7]).

**Figure 1:**
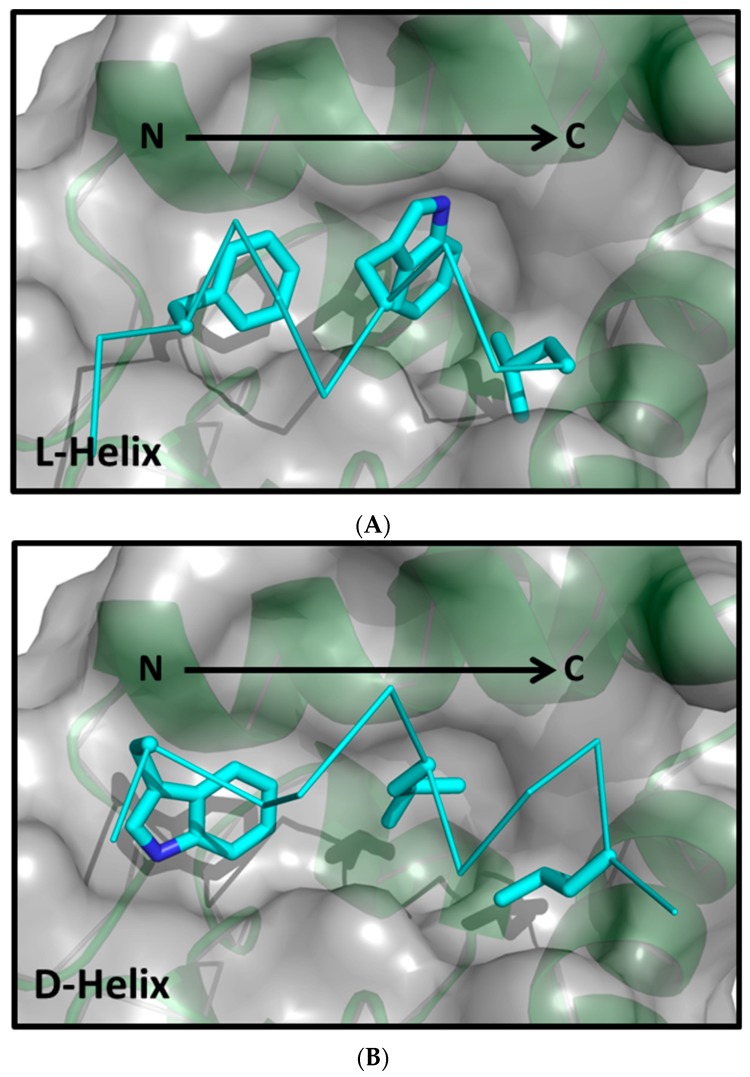
A comparison of the critical binding element of an L-α-helical peptide compare to those of a D-α-helical peptide. (**A**) The L-helical peptide (3jzs [4]). (**B**) The D-helical peptide (3lnj [8]). The preferred residues of the L-α-helix for the three critical pockets are Phe, Trp and Leu whereas the three corresponding interactions of the D-α-helix are Trp, Leu and Leu. In both cases MDM2 is shown in the same orientation.

A noteworthy MDM2/MDMX-targeted SAR dataset with peptide ligands comprised of all D-α-helices is provided by Liu and coworkers [8]. In this work, mirror image phage display was used to find an all D-peptide, referred to as PMI-α, that binds potently to MDM2. Importantly, there are three MDM2 crystal structures with all D-amino acid α-helical peptides (3iwy [8], 3lnj [8] and 3tpx [9]). Interestingly, the SAR for the all D-amino acid α-helices differs markedly from that of the L-amino acid α-helices. For example, while the same three MDM2/MDMX pockets are filled by three critical residues of each peptide, the specific nature of the residues is quite different: F-W-L for the L-α-helices and W-L-L for the D-α-helices (Figure 1). Further, the L-α-helices described in the previous paragraph lose 2–5 fold in binding affinity for MDMX relative to MDM2 whereas the D-α-helical peptides lose nearly 100–1000 fold in binding affinity [9].

A significant MDM2/MDMX-targeted α-helical peptide SAR dataset was developed using stapled α-helical peptides. Stapled α-helical peptides are engineered by chemically connecting two side-chains (*i* and *j*) in a helix, typically using an alkyl chain, which can result in stabilization of the α-helical conformation. There are five MDM2/MDMX structures with bound stapled α-helical peptides: (1) 3v3b [10] (1–7 stapled α-helix bound to MDM2), (2) 4ud7/4ue1 [11] (1–4 stapled α-helix bound to MDM2), (3) 4umn [12] (1–7 stapled α-helix bound to the M62A MDM2 mutant), (4) 4n5t [13] (the 1–7 stapled α-helix ATSP-7041 bound to MDMX), and (5) 5afg [14] (a more complicated 1–7 stapled α-helix bound to MDM2). These structures come with excellent SAR, including investigations into the effects of different staples on p53 [15], the impacts of a number of single residue changes, and a full alanine scan around the stapled L-α-helix ATSP3900 [13,16].

In addition to the structures with stapled α-helices, there is a single structure, 3iux [17], with MDM2 bound to a bicyclic peptide with two disulfide bonds, referred to as stingin. Also, there is a single structure of a non-α-helical peptide bound to MDM2, 2axi [18]. This peptide is a 10 residue N–C cyclic peptide adopting a β-hairpin conformation which is capped by a Pro-D-Pro motif. This data set is remarkable in that it includes MDM2 binding data for >70 analogue peptides, many of which contain non-natural amino acids in addition to the invariant D-Pro.

In what follows, we describe the use of several of the computational tools that comprise our integrated computational peptide design platform (CMDInventus) to investigate the extent to which they can reproduce the aforementioned p53/MDM2/MDMX SAR data and prospectively predict the binding effects of conservative and non-conservative peptide ligand modifications. Toward this end, we first describe the use of our physics-based conformational sampling algorithm to successfully model and reproduce the observed bound conformations of several peptide ligands. Second, we describe the utility of our binding free energy estimation methods to reproduce the measured binding and activity trends present in the various data sets. Importantly, all retrospective or post-data collection calculations were performed without explicit fitting or re-parameterization of any kind. Of course, the most demanding test of any method is its accuracy at predicting future observations. Hence, we describe the use of the various computational methods to model and prospectively calculate or blindly predict prior to data collection binding affinities for two series of peptides, one corresponding to an alanine scan and the second to a D-amino acid scan of a stapled α-helical peptide. The prospective all-D scan study is of special importance, as it is experimentally novel and resulted in some nonintuitive computational predictions being confirmed by experiment.

## 2. Results and Discussion

Because a typical CMDInventus peptide modeling and design work-flow integrates the use of CMDpeptide, CMDescore, CMDyscore and CMDboltzmann, we deemed it important to test all approaches wherever possible in retrospective and prospective analyses. The strength of the retrospective analysis is that it allows the methods to be tested against a large and diverse amount of data. The problem with retrospective testing, however, is that it may suffer from various biases. It is hoped that prospective testing will help mitigate these biases. By testing methods retrospectively and prospectively, it is hoped that information will be gained about the role each method can play in a CMDInventus design work-flow.

### 2.1. Retrospective Study

**Figure 2:**
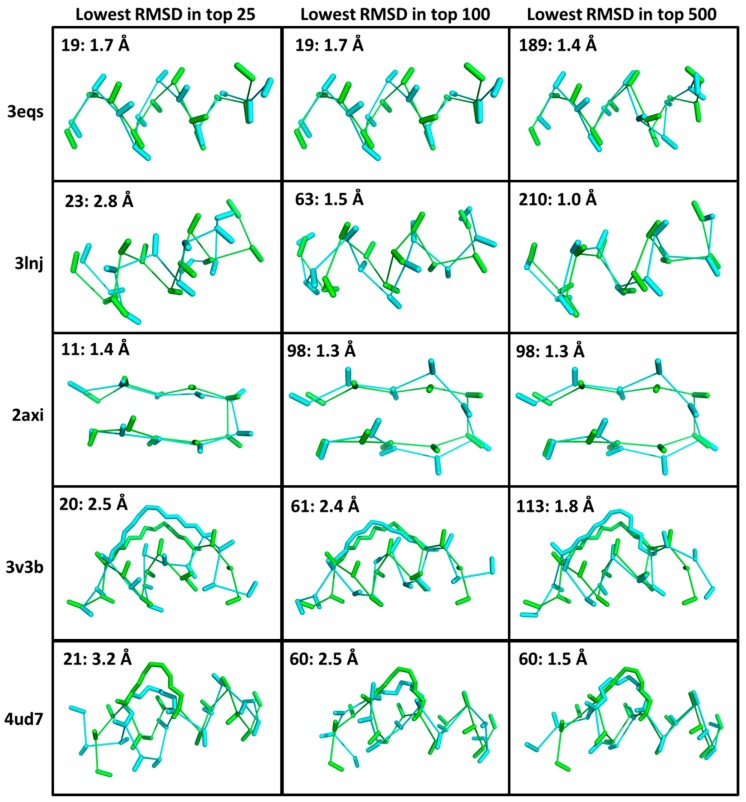
Selected conformations from CMDpeptide calculations. Here, we show select results from Table 2. For this figure, we took the peptides from the PDB structures below. The first column of images shows the conformations in the top 25 ranked conformations with the lowest RMSDs to the bound conformations. The second column of images shows the conformations in the top 100 ranked conformations with the lowest RMSDs. The third column of images shows the conformations in the top 500 ranked conformations with the lowest RMSDs. In each case a conformational ensemble was generated starting from a sequence using the default parameters of CMDpeptide. In each case, the bound conformation is shown in green and the CMDpeptide conformation in cyan. Each peptide is shown as a ribbon with the C_α_-C_β_ bond. Additionally, for the stapled helices the staple is shown.

Conformational sampling calculations with CMDpeptide. CMDpeptide calculations start from peptide sequences and, when appropriate, inter-residue bonding information such as disulfide bonds. In particular, they do not employ any structural information. The results of the peptide conformational analysis are shown in Table 2 and Figure 2. All RMSDs reported are calculated over the backbone atoms and C_β_ atoms. This provides a more demanding measure of structural similarity than does the more typical RMSDs over just C_α_ atoms because including the C_β_ atom captures side-chain orientation. An assumption behind CMDpeptide is that calculated ensembles will include a broad range of biologically relevant peptide conformations, including protein binding conformations. As will be shown below, this assumption is validated by our results.

In all cases studied, there is at least one peptide conformation among the low-energy conformations (within 15 kcal/mol of the lowest energy conformation) within an RMSD < 1.6 Å of the co-crystallized peptide ligand conformation with an overall average RMSD of 1.12 Å. In fact, for seven of the sixteen cases conformations with RMSDs < 1.0 Å were identified. Hence, CMDpeptide can be used to consistently sample conformations close to the bound conformation observed in MDM2/MDMX cocrystal structures. This is an encouraging result, especially given that a range of peptide lengths (8–18 resides) and chemical types (L-linear, N–C cyclized, bicyclic, D-linear, and L-stapled) were studied and that much of the calculated RMSD values result from conformational deviations at N and C termini. Similarly, it is worth pointing out that there is a relatively weak but non-trivial correlation between peptide length and best low-energy RMSD (R ≈ 0.46). In fact, a simple two term regression model that includes peptide length and the presence or absence of a staple as independent descriptors can be used to account for ≈ 68% of the measured variation in lowest RMSD.

Not surprisingly, the average best peptide conformation RMSD trends down from 2.38 Å, to 2.16 Å, to 1.48 Å as the number of as the number of conformations allows increases from 25, to 100, to 500. Importantly, even at 500 clusters the best low-energy RMSD peptide identified is never present. So, while the results are encouraging, they suggest that the improvements in the scoring strategy are still needed for identifying and ranking best RMSD peptide binding conformations. This conclusion is reinforced by the fact that our current force field scoring and clustering protocols failed to pick out best RMSD conformations where sampling does not appear to be an issue (sampled conformations within ≈ 0.8 Å of X-ray). It remains a possibility, however, that incomplete sidechain sampling failed to identify a sidechain interaction that would have led to the ranking of those conformation close to the X-ray conformation near to the top of the ensemble. Indeed, the combination of the large sampling space with the scoring problem makes the peptide conformational analysis a difficult challenge.

**Figure 3:**
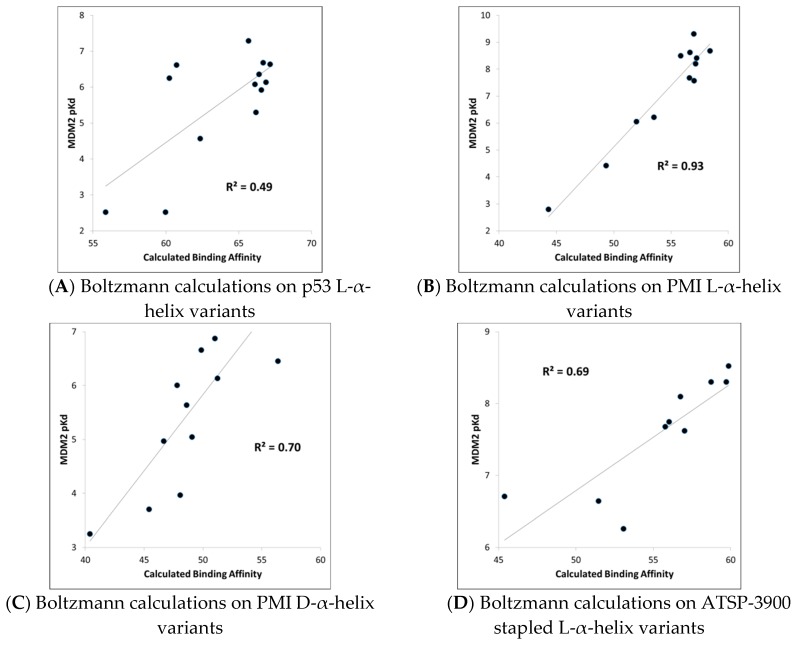

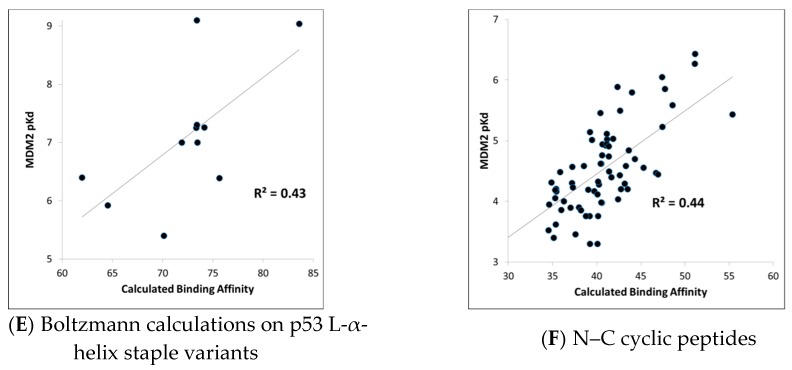
The CMDboltzmann calculations on the individual data sets—All figures show CMDboltzmann calculated binding affinities recorded on the X-axis and experimental binding affinities (typically pKd) recorded on the Y-axis. (**A**) Structure activity relationship (SAR) around the L-α-helix p53 [4]. (**B**) SAR around the L-α-helix PMI [4]. (**C**) SAR around the D-helix PMI-α [8]. (**D**) SAR around the stapled L-α-helix ATSP-3900 [16]. (**E**) SAR around L-α-helix p53 stapled variants SAR [15]. (**F**) SAR around the N–C cyclic peptide from the 2axi structure [18].

MDM2-peptide binding affinity calculations with CMDboltzmann. As described in detail in the Materials and Methods section, CMDboltzmann is a tool for estimating the binding affinity of a peptide given knowledge of the binding mode of a portion of its backbone. It particular, it locally samples a peptide conformation in the binding site of a protein producing a calculated binding affinity that is based on numerous similar binding modes of the peptide. As a result, it tends to be less sensitive to the exact details of the starting binding mode. The results of the CMDboltzmann calculations on the various data sets are shown in Figure 3. In all, six MDM2/MDMX SAR datasets were analyzed: (1) p53 L-helix variants, (2) PMI L-α-helix variants, (3) PMI-α D-α-helix variants, (4) ATSP-3900 stapled L-α-helix ala scan variants, (5) p53 L-α-helix staple variants, and (6) N–C cyclized variants. Hence, the CMDboltzmann binding affinity calculations cover diverse peptide chemistries and topologies. The CMBboltzmann calculations were all performed using the 3eqs structure of MDM2 using the binding mode extracted from the corresponding co-crystal structures with the respective peptide.

As can be seen from Figure 3, the R^2^s between calculated and experimental binding affinities vary from 0.43 to 0.93. Hence, the CMDboltzmann calculations can account for some 43%–93% of the measured binding affinity variation. This is an encouraging result, especially given the diverse sizes and topologies of the peptides studied and the large changes in amino acids found in the data sets particularly the N–C cyclized variants.

**Figure 4:**
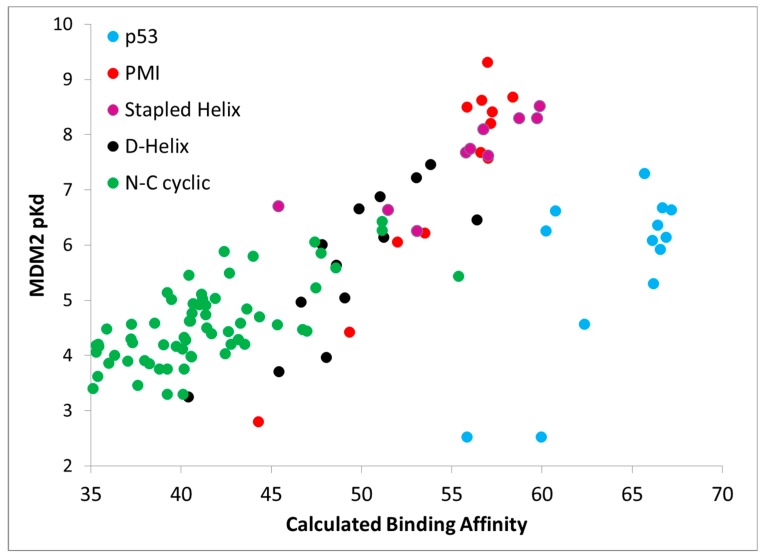
Boltzmann calculations on the entire MDM2 data set. Each of the four data sets shown in Figure 3 above is plotted as a separate color. As is apparent the Li-p53 data set stands out as having higher than expected calculated binding affinities in comparison to the other data sets. There are two reasonable explanations for why the affinity of the p53 analogs is generally overestimated relative to the rest of the peptides. First, these peptides are highly negatively charged while MDM2 has a net positive charge and the empirical solvation model used may not be adequate to address the difference in charge. Second, p53 is known to be highly disordered in solution thereby encurring a large free energy penalty upon binding in the α-helical conformation whereas the other peptide families are known to be more stable and structured in solution.

Further, Figure 4 shows calculated binding affinities versus measured pK_d_ values for all peptides studied, color coded by data set. Once again, the results are encouraging, as a fairly straight line can be drawn through the calculated and measured binding affinities for all of the PMI L-α-helix, PMI-α D-α-helix, ATSP-3900 stapled L-α-helix, and N–C cyclized peptides. With just these four data sets, i.e., omitting the p53 peptide analogs, the overall R^2^ is 0.75. By including the p53 peptide analogs, the R^2^ falls to 0.48. The p53 peptide analogs show higher calculated binding affinities than expected, suggesting that a penalty contribution of some kind is missing from the CMDboltzmann model. The missing penalty contribution likely derives from two missing factors. The first factor is the inability of the solvation model to adequately counter balance the electrostatic binding interactions. P53 is a highly negatively charged peptide, as it contains four charged residues and both termini are free and ionized, and the high charge may pose a serious challenge to the EEF1 solvation model. In essence, CMDboltzmann overestimates the electrostatic contributions from charged amino acids. The second missing factor has to do with the conformational penalty associated with the p53 peptide “climbing” into its binding conformation. Indeed, p53 and direct analogs are known to be disordered in water and only adopt an α-helical conformation in the context of MDM2/MDMX binding sites. In fact, p53 has been shown to be approximately 11% α-helical in solution [15]. By comparison, PMI has been shown to readily adopt an α-helical conformation in water as well. Hence, it is reasonable to assume that p53 will suffer a greater free energy cost upon binding when compared to the other peptide ligands. Thus, future work might focus on improving the CMDboltzmann electrostatic solvation model and extending CMDboltzmann to include sampling of unbound conformations to better estimate the free energy penalties associated with burying charged groups and flexible ligand binding.

**Figure 5:**
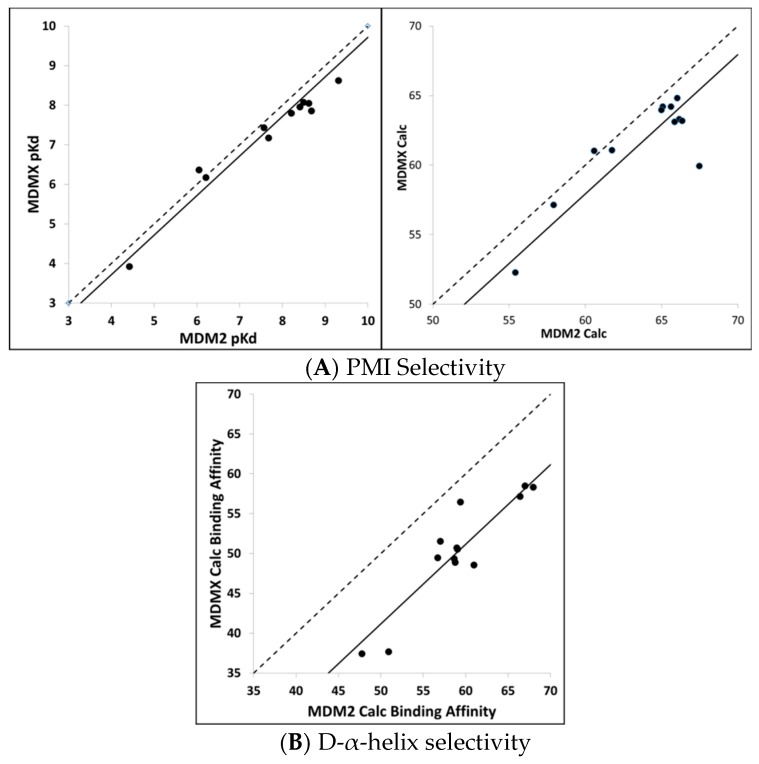
Selectivity Calculations with CMDboltzmann—Here, we compare measured MDM2/MDMX selectivity to calculated selectivity. (**A**) The left panel shows the experimental pKd of MDM2 versus the experimental MDMX pKd for PMI analogs [4]. The dashed line shows the line where the MDM2 and MDMX are identical. The solid line shows the best fit line to the MDM2/MDMX data. Clearly, the MDM2 and MDMX binding affinities are very close to one another. The right panel shows the comparison of the MDM2 and MDMX calculated binding affinities for the same peptides. Again, the dash line shows where the MDM2 and MDMX calculated binding affinities are identical and the solid line is the best fit between the two. In the calculated case, we see a slight preference for MDM2 over MDMX. (**B**) This panel shows a comparison of the MDM2 and MDMX calculated binding affinities for the D-α-helical analogs [8]. Here, we see a large preference for MDM2 over MDMX. In this case, the experimental binding affinities are not known for all of the peptides. It is known, however, that the two that are most potent for MDM2 are much weaker for MDMX [9]. Thus, the selectivity calculations are in encouraging qualitative agreement with the available experimental data for the D-α-helical peptides as well.

Calculating MDM2/MDMX binding selectivity with CMDboltzmann. As a final test of the CMDboltzmann procedure for estimating binding affinities, we examined the differences in MDM2 and MDMX binding affinities. The MDMX calculations were all performed using the MDMX structure from 3jzo [19]. For the PMI analogs [4] and L-α-helices the differences in binding affinities are modest. In particular, on average the PMI analogs show two-fold more affinity for MDM2 over MDMX (see Figure 5). Encouragingly, with the exception of a single peptide, our calculations qualitatively agree with experiment as they show very small differences between calculated binding affinities between PMI and MDM2 and MDMX. In contrast, the two most potent of the D-α-helical peptides have been shown to be essentially inactive versus MDM2 [9]. As can be seen in Figure 5, we find substantial differences between the calculated MDM2 and MDMX binding affinities for the D-α-helical peptides. Thus, the MDM2/MDMX affinity selectivity calculations are in encouraging qualitative agreement with the available experimental data for the D-α-helical peptides as well.

Calculating binding affinities from single structures using CMDescore and CMDyscore. CMDescore and CMDyscore, as described in the Materials and Methods section, score peptide binding modes based on a single fixed complex structure. All CMDescore and CMDyscore scoring methods were used to calculate MDM2/MDMX-peptide binding affinities for the datasets summarized in Table 3. For comparative purposes, the Vina and X-score scoring functions and simple surface area and packing empirical functions were also used on the same datasets. Binding affinity and binding energy calculations are shown either ignoring inactive peptides or accounting for them by giving them a very low binding affinity with results summarized in Table 3A,B, respectively. The latter only affects the Li p53 data, but adds information on the ability of the methods to distinguish good from very poor binders. Hence, our focus will be more focused on analyzing the results from Table 3B.

As indicated in Table 3B, CMDescore generated useful binding affinity estimates for all seven datasets, resulting in an average R^2^ of ≈ 0.69. This is an encouraging result, especially given its simplicity, physical intuitiveness, and rapid speed of calculation. Comparing the various CMDyscore results (Table 3B), one can see the clear benefit of adding a solvation term to the force field. Indeed, CMDyscore, when combined with solvation, posted some good correlations and, with two exceptions, consistently outperformed all other scoring procedures resulting in an impressive average R^2^ of 0.81. Using uncharged amino acids with CMDyscore resulted in an average R^2^ of ≈ 0.73. The simple implementation of CMDyscore produced an R^2^ of ≈ 0.62. This pattern suggests the hypothesis that mitigating the impact of charged residues on binding is of considerable importance and that, in the present study, this is best achieved with an implicit solvation model. Our attempt to consider strain in the bound conformation with CMDyscore calculations resulted in the poorest average CMDyscore result (R^2^ ≈ 0.60).

It is interesting to note how well the simple packing scoring function performed, yielding an average R^2^ of ≈ 0.75 (Table 3B). The packing score is a quality metric available in YASARA [5]. It is a weighted average of three individual metrics: normality of dihedral angles (0.145), normality of 1D distance-dependent packing interactions (0.390), and normality of 3D direction-dependent packing interactions (0.465). The metrics are knowledge-based potentials. The dihedral potential is based on the probability of finding the observed dihedral in a reference PDB database. The 1D potential is based on probabilities of finding specific atom–atom distances. The 3D potential is based on probabilities of finding atoms in a specific direction where the coordinate system is based on a central heavy atom and two of its bonded neighbors. All probabilities are converted to energies which are then converted to Z-scores. Perhaps this is not too surprising for the MDMX and MDM2 systems, as binding to both targets is dominated by the three hydrophobic hot spot residues. Moreover, success for a simple packing scoring function reproducing affinity trends for known binders to known interfaces does not entail success at predicting hypothesized binders to hypothesized interfaces—a key aspect of computational peptide drug design—where tight packing can be offset or reinforced by electrostatic and desolvation effects, etc. Considerations like these caution against generalization and justify the continued development and use of more complicated multi-term scoring functions, even when correlations prove to be similar on specific datasets.

Also, worth commenting on is that the scoring functions provide quantitative binding affinity values for all peptides, whereas in the Li dataset some peptides are only listed as qualitatively ‘inactive’. In order to include information from inactive peptides, the K_d_ for these peptides was set to −2.0 kcal/mol. A comparison of the results summarized in Table 3A,B shows that this addition significantly affects the calculated R^2^ values for the p53 dataset, illustrating that it is much easier for a scoring function to determine active from inactive compounds than it is to quantitatively discriminate among a set of active compounds.

**Figure 6:**
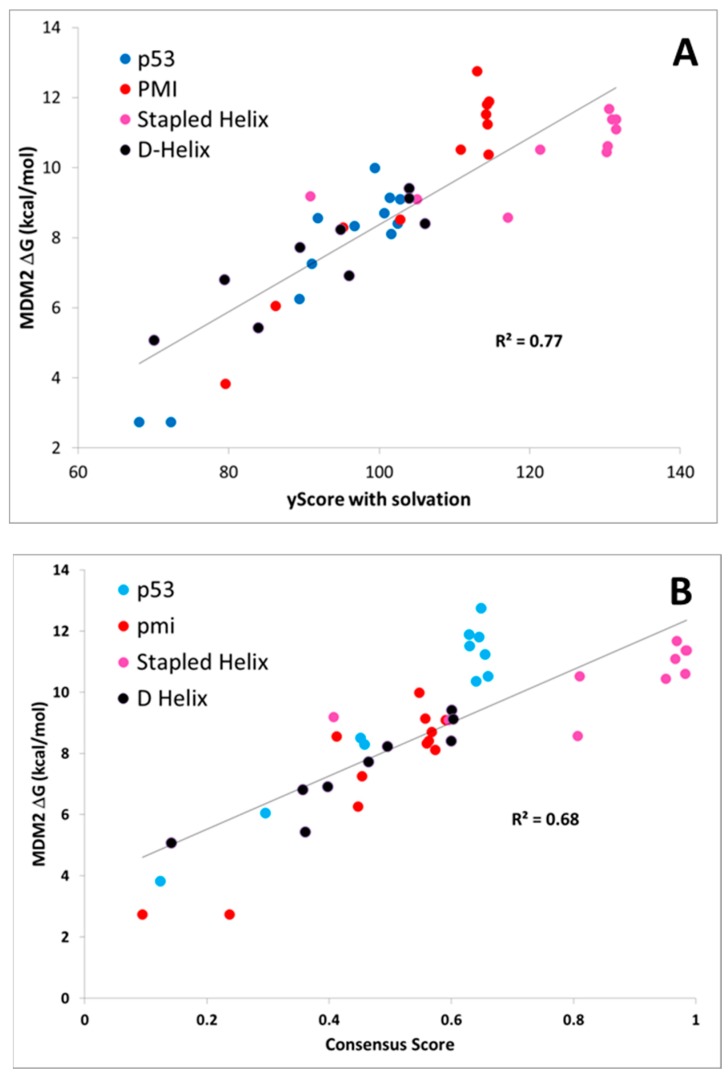
Single pose scoring results with CMDyscore with solvation (**A**) and the consensus score (**B**).

A plot of the PMI, p53, D-α-helix, and stapled α-helix predictions for the best single scoring function (CMDyscore with solvation) and a consensus scoring function are shown in Figure 6. The consensus score was created by combining the predictions of CMDescore, CMDyscore, VINA, and Xscore. For each score, the values were normalized between zero (worst score) and one (best score). The consensus score then was taken as the mean of these four normalized scores. This consensus score was then plotted against the normalized binding energies. In the case of MDM2, the best single function outperformed the consensus. For MDMX, however, the consensus score proved to be superior. Note also that the overall correlation across the data sets is not as strong as with any individual dataset. This is not surprising, given the data was collected by different groups.

**Figure 7:**
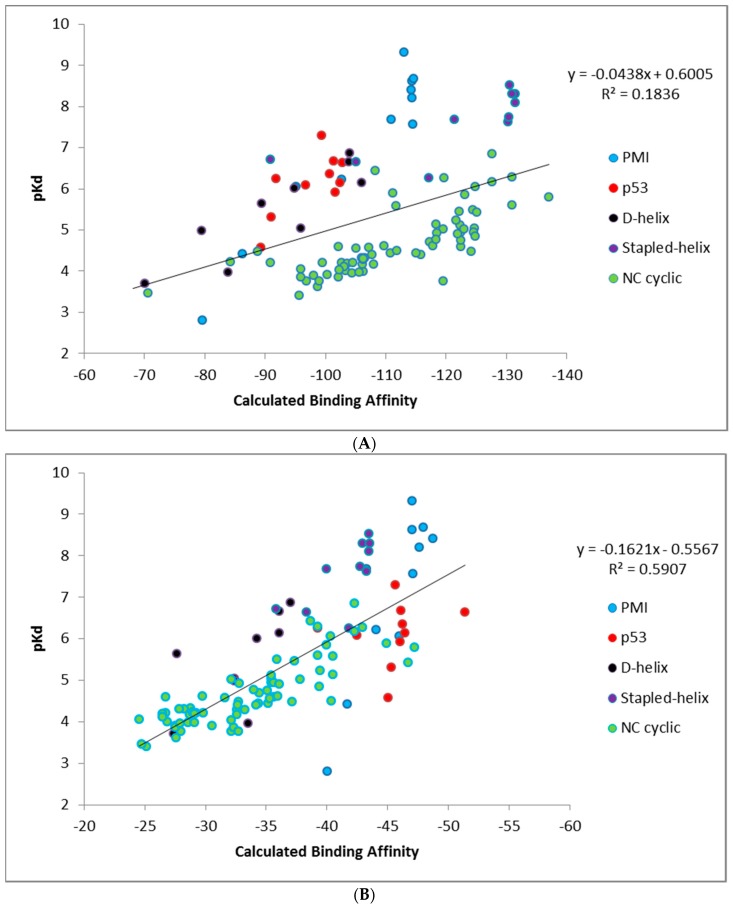

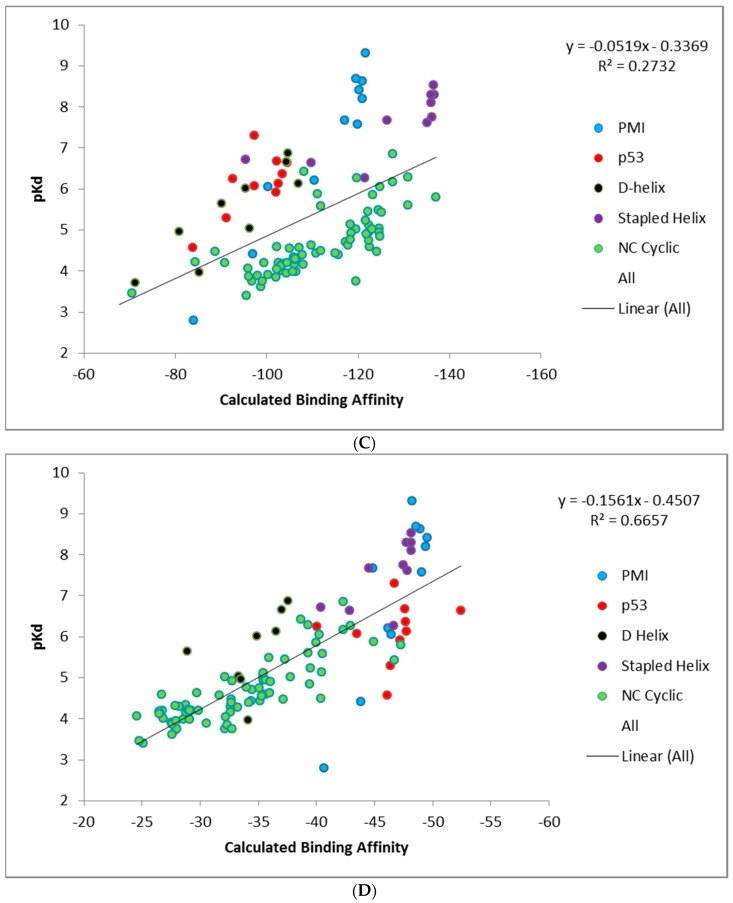
CMDyscore with solvation on the full data set. (**A**) CMDyscore with solvation with default CMDyscore settings. (**B**) CMDyscore after more extensive minimization of all datasets using solvation (**C**) or default settings (**D**).

Unlike CMDescore, CMDyscore is parameterized for non-standard amino acids. Hence, a plot of the CMDyscore results with N–C cyclized mutants included was also prepared and is provided in Figure 7. Including the cyclic peptides, with the primarily alanine, scanning results of the other datasets (PMI, p53, D-α-helix, and stapled α-helix), adds a number of complications for making realistic comparisons using single point calculations. One complication is that for the earlier calculations, only a single residue was mutated and, in general, the mutation was to a residue smaller in size than the original. All the cyclic peptides were built from a single starting template and multiple mutations involving both larger and smaller residues were required. As a result, more extensive minimization of the starting structure was carried out. The cyclic peptides also involved more variation in the number and type of charged residues.

Figure 7 summarizes the CMDyscore results with the combined datasets. It is apparent in Figure 7A that despite reasonable correlations in the individual datasets, cyclic peptides are shifted to the right with respect to the other datasets, i.e., their calculated affinities are over estimated relative to the helical peptides. In the combined dataset, the default CMDyscore parameters show good correlation with the combined data set. It was thought that part of the problem was that different protocols were used to minimize the complexes. All complexes were therefore run through another series of minimizations and rescored. First all but the peptide side chains were fixed and the complex minimized. A second minimization step was done allowing all side chains to relax. Finally, a third minimization was done with the protein fixed and the peptide completely free. The correlations did improve. The R^2^ for CMDyscore with solvation improved from 0.18 to 0.27. More impressive was the performance of the default CMDyscore, which improved from an already respectable R^2^ of 0.59 to 0.67. To a large extent, this is because the default method better predicts the large cyclic peptide data set, but one can also see from the graph for the default method, that the cyclic peptides are no longer shifted to the right.

The most likely reason for the observed results is that the PBSA in YASARA uses a somewhat ad hoc value for scaling the surface area. When used on data sets of similar size and composition, it improves the correlation. For a more diverse data set it is not helpful. The results may improve by better parameterization of the SA term. However, the focus of the article is on a rigorous examination of the existing methods and exploring methods to improve the results after the fact is outside the scope. Nevertheless, mutation and single point energy calculations following a mild minimization protocol do provide reasonably good correlation with experiment.

### 2.2. Prospective Study

In addition to the retrospective calculations, the same methods were used to prospectively estimate or to blindly predict the binding affinities of a series of analogs of ATSP-7041 (A8Q, Q9L) to MDM2. In particular, the calculations were performed on an Ala scan and novel D-amino acid scan of an ATSP-7041 analog (having A8Q and Q9L modifications) prior to synthesis and experimental binding affinity measurements. Undoubtedly, putting blind predictions in jeopardy of observational falsification provides a more rigorous test of a computational method.

**Figure 8:**
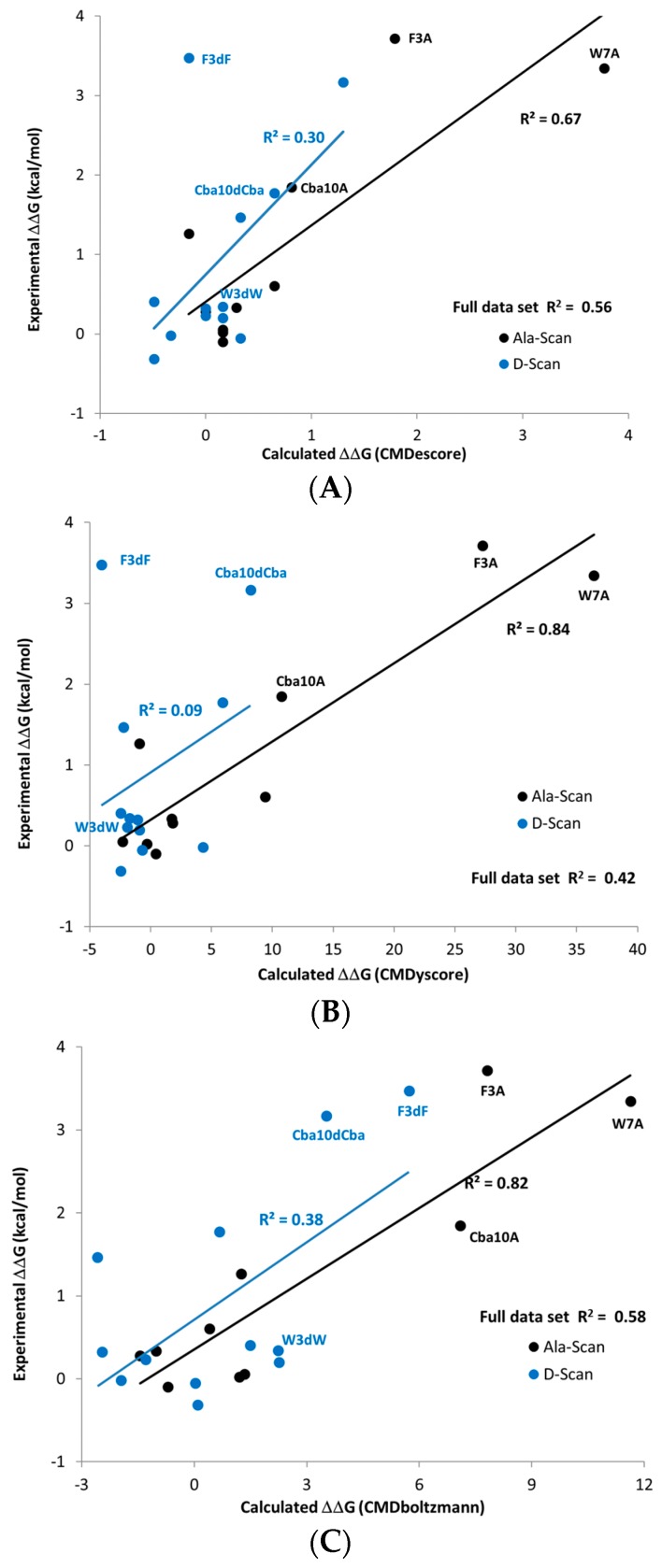
Scoring results on the prospective data. (**A**) CMDescore. (**B**) CMDyscore (**C**) CMDboltzmann.

The predictions made using the sampling-based CMDboltzmann method and the single pose scoring functions CMDyscore and CMDescore are shown in Table 4 and Figure 8. Importantly, all three computational binding affinity prediction methods produced generally good agreement with the experimentally measured Ala scan data of the ATSP-7041 analog binding to MDM2, with the R^2^s for CMDescore, CMDyscore and CMDboltzmann being 0.67, 0.84 and 0.82, respectively.

Surprisingly, all three methods predicted that the mutation of Trp7 to dTrp7 would lead to only a modest drop in potency. This prediction was eventually confirmed by experimental measurements. Interestingly, CMDescore and CMDyscore were used to predict that the Phe3 to dPhe3 mutation would have a small effect, while the application of CMDboltzmann predicted a strongly destabilizing effect. The CMDboltzmann prediction was later confirmed by experimental measurement. Subsequently, a crystal structure, 6aaw [20], was solved further validating the fit of the dTrp sidechain. The differential prediction successes of CMDescore, CMDyscore and CMDboltzmann on the Phe3 and Trp7 mutants helps to account for the R^2^ values and trend obtained for the full MDM2-ATSP7041 D-amino acid scan results (0.30, 0.09 and 0.38 for CMDescore, CMDyscore and CMDboltzmann, respectively).

When Phe3 is mutated to dPhe3, the aromatic rings of the two cases overlap almost completely. It is thus not surprising that the empirical CMDescore scoring function displays problems here, as the number of carbon–carbon contacts is identical. The dPhe3 mutation does result in short van der Waals contacts with two residues in the protein, which ideally a physics-based force-field would identify as unfavorable. As part of the CMDyscore binding energy calculation, however, the peptide was fully minimized in the binding site which may have removed the bad contacts, resulting in a failed prediction. Interestingly, the correlation obtained with CMDyscore without the minimization step is significantly better (0.74) and still predicts that a mutation to dTrp will have little effect. The differences between the minimized and un-minimized predictions might be because the strain in dPhe3 is propagated to the backbone upon minimization, where it will not have an effect on a single point binding energy calculation. It must be admitted, however, that all of this may be coincidence, as the un-minimized correlation result is not the result of a blind study. It does, however, suggest that generating affinity predictions from a conformational ensemble, as is done with CMDboltzmann, captures more subtle structural effects and can be worth the extra computational expenditure. The sub-optimal R^2^ obtained with CMDboltzmann on the D-scan data stems from the Thr2 to dThr mutation, where the measured drop in potency is just over a log unit whereas the CMDboltzmann prediction is of a slight increase in binding affinity. Recall that the CMDboltzmann procedure allows the two residues at the N- and C-termini to be fully flexible (they are not constrained whatsoever). Indeed, this might have allowed too much flexibility to be incorporate into the simulation, resulting in the poor Thr2 to dThr2 prediction results.

In summary, the Ala scan data of the ATSP-7041 analog (see Table 4) closely mirrored that of ATSP-3900 [16], with large losses in potency for any of the F3A, W7A or Cba10A mutations and modest to no loss in potency when other residues are mutated to Ala. Importantly, CMDescore, CMDyscore and CMDboltzmann were used to generate Ala scan predictions that were ultimately shown to agree nicely with experimental measurements.

The corresponding D-amino acid scan of the ATSP-7041 analog, however, produced an experimental surprise, namely that the change of Trp7 to dTrp7 led to a small drop in binding affinity. Based on Figure 8C it is apparent that the drop in calculated affinity with CMDBoltzmann for W7dW is within the range of the majority of other changes that led to relatively small experimental affinity changes. On the other hand, the changes F3dF, F3dA, W7A and Cba10A all showed large drops in calculated and experimental affinity. Finally, CMDboltzmann showed a modest drop in calculated affinity for the Cba10dCba change whereas the experimental drop in affinity was comparable to that of the F3dF, F3dA, W7A, and Cba10A changes. While the change in CMDescore and CMDyscore, Figure 8A,B respectively, is indeed small for W7dW, these scoring functions also do not change with the F3dF and Cba10dCba changes. This highlights the value of including the computationally more demanding sampling when making more complex changes. Indeed, the poorer agreement with the d-Amino Acid scan when compared to the Ala-scan may in part be to a larger difference in the solution behavior when the amino acids are change to their D counterpart.

Comparison of Single Point Results with CMDBoltzmann Results. In general, calculation of relative binding affinities using either a single point low-energy conformation or an ensemble of low-energy conformations and an appropriate scoring function has proven fairly effective for the MDM2/MDMX target. The single point calculations have the advantage of being fast, allowing screening of large numbers of peptides. The ensemble-based approach has the advantage of more fully defining the conformational space of the peptides and appears more robust. The single point method, if not given an appropriate starting conformation, can fail completely. No one conformation is likely to dominate in the Boltzmann ensemble.

Importance of solvation model to adequately complement electrostatics. A good solvation model should account for two situations which are not adequately represented using a simple ‘gas-phase’ implementation of a force field. The first is the affinity gain from associating hydrophobic surfaces in the complex and the affinity loss from desolvating polar groups involved in binding. The second is a better treatment of attenuation of charge–charge interactions in a polar medium. When molecular dynamics is used to calculate an ensemble of low-energy conformations, the overall charge of the system can be neutralized by the addition of counter ions and explicit solvent can be used to attenuate the Coulombic interactions. One challenge of accurately scoring protein–peptide interactions is that it is not feasible to include counter ions or explicit solvent when comparing the energy of two protein–peptide complexes. One thus needs to handle the problems of charge neutralization and attenuation using more ad hoc methods.

In this study, several techniques were used. Neutralizing the charged groups was tried as a method of both obtaining charge balance and attenuating Coulombic interactions. The problem with this approach is that ionic interactions become hydrogen bond interactions. An alternate approach was used in CMDyscore. In that case, charges were adjusted so as to provide an overall neutral complex and the PBSA method was used to account for polar and nonpolar solvation terms.

To handle attenuation of the Coulombic interactions several methods were used: scaling the charges, using a distant-dependent dielectric constant and using a PBSA implicit solvent model. Comparing CMDyscore variations, the simplest approach (scaled charges) shows poorer correlation with experiment than either using neutral groups or the PBSA solvation model. The best average correlation on the linear helical data was achieved when using the PBSA implicit solvent model. The reason may be the more accurate treatment of the hydrophobic effect. The MDM2/MDMX system is perhaps not the best test for methods for implicitly handling charges. The binding is entirely due to hydrophobic interactions with the charged residues largely being solvent exposed. We have found that in general that implicit solvation models have problems when there are multiple charged groups not involved in binding. These charged groups should be balanced by a nearby counter ion, but in an implicit model, they are not. The summation of these unbalanced long-range interactions can significantly affect the calculated energy, distorting the results. Thus, correlation of binding affinity with calculated binding energy in our experience is much better with peptides that primarily consist of polar and nonpolar amino acids, as is the case with MDM2/MDMX.

dTrp7 results. The comparison of the predictions for dPhe3 and dTrp7 are particularly interesting. At first glance, the D and L conformations are quite similar. In both cases, the aromatic rings overlap almost completely. In essence, the β-carbon is either above or below the plane of the backbone, with the aromatic rings angled into the same region of space.

In the case of Trp7, there are two features which distinguish it from Phe3: the tryptophan makes a hydrogen bond to Met50 in the protein (the Trp N are almost exactly superimposed) and the pocket containing the Trp7 is not quite as tight as for Phe-3. In the single point (minimized) mutations the larger dTrp has six contacts closer than 3 angstroms, the closest being 2.1 angstroms, while dPhe has seven contacts, the closest being 1.6 angstroms. In addition, when the complexes are aligned on the protein, the C alpha carbons in Trp7 and dTrp7 are 0.57 angstroms apart, whereas the C_α_ carbons in Phe3 and 0.94 angstroms apart. These are subtle differences and it is remarkable and highly encouraging that CMDBoltzmann was able to detect the difference and correctly predict that only Trp7 mutating to dTrp would not show a significant loss in affinity.

Implications for CMDInventus work-flow approach to peptide modeling and design. Our approach to computational peptide drug design employs multiple methods in a hierarchical and integrated fashion. In a typical work-flow, CMDpeptide can be used to model the solution and binding conformations of a peptide scaffold which can then be systematically mutated and evaluated for its binding affinity to a protein target using methods that range from computationally fast and un-rigorous (CMDescore) to slower and more rigorous (CMDyscore) to very slow and highly rigorous (CMDboltzmann). Hence, all three binding affinity prediction methods (and by implication CMDpeptide, as it is used in CMDboltzmann calculations) were tested retrospectively (using previously published data) and prospectively (using novel data collected after computational predictions had been made).

The results presented here validate the use of CMDpeptide to model biologically relevant conformations for diverse MDM2/MDMX peptides, as it yielded X-ray like conformations for all cases tested. Future work will focus on developing an improved scoring strategy for identifying relevant conformations from calculated conformational ensembles. Also, while CMDpeptide was indirectly tested in the prospective CMDboltzmann study, a direct test of CMDpeptide in a prospective study would also be desirable.

Our results also validate the integrated use of CMDescore and CMDboltzmann without the need for re-calibration, as both produced consistently encouraging results across multiple data sets in both the retrospective and prospective studies and with CMDboltzmann producing overall better results than CMDescore. Having said that, additional research is needed to parameterize and test CMDescore on the 2axi cyclized multiple mutation data asset and for use with non-standard amino acids more generally. Similarly, more research is needed to develop an optimized force field and solvation model for use with constrained CMDboltzmann (and CMDpeptide) dihedral space Monte Carlo and minimization calculations. CMDyscore with solvation produced the best overall results in the retrospective analysis of the linear peptide/helical datasets. To obtain good results on the multi-mutation cyclized 2axi data, however, required the use of a more extensive minimization protocol or the substitution of the default CMDyscore for CMDyscore with solvation. Similarly, CMDyscore with solvation produced nice results in the alanine scanning prospective study but poor results in the prospective D-scanning study that were improved ad hoc by eliminating any minimization prior to scoring. This suggests that the best CMDyscore flavor and minimization procedure should, whenever possible, be calibrated, selected and applied on a case-by-case basis. In the absence of training data, it seems the best choice would be CMDyscore with solvation using the standard minimization protocol. Future work will focus on developing a more generally useful flavor of CMDyscore or on developing a general and systematic procedure for selecting the best flavor of CMDyscore to apply to a particular protein–peptide system.

The results presented here suggest a robust role for CMDinventus in a structure-based drug design project. Given the vast sequence space accessible around a binding mode for even a modestly sized peptide, fast simple scoring functions such as CMDescore and CMDyscore are necessary. Particularly, by considering side-chain changes one at a time, these scoring functions can be used to rapidly search 1000s of possible side chains per position ultimately narrowing the list to a handful that are the most promising. Depending on computing resources, CMDboltzmann can be used to further and more accurately prioritize 100s to 1000s of the sequences of greatest interest. Indeed, the results of these calculations could be used to prioritize further synthesis or could be used for more computationally expensive methods such as molecular dynamics [21,22], free energy perturbation [23,24] or thermodynamic integration [25,26].

## 3. Materials and Methods

CMDInventus is a proprietary platform for computer-aided peptide drug design. CMDInventus consists of self-contained computational tools or modules for solving specific biophysical problems. The various modules can be strung together and integrated to form project-specific peptide drug design work-flows. The present study focused on the use of several CMDInventus modules to run retrospective and prospective calculations for various MDM2/MDMX datasets.

All retrospective and prospective calculations either involved explicit sampling of peptide conformational space and/or configurational (conformational + translational + rotational) space or were derived from single crystal structures. In the present study, we employed two modules that use the same code base for peptide conformational and configurational sampling. The first application, called CMDpeptide, is for physics-based peptide conformational sampling from sequence information alone. Here the goal is to start with a peptide sequence, and where applicable inter-residue bonding information, and generate an ensemble of three-dimensional peptide structures that contains all biologically relevant conformations. The ensemble of conformations should include those relevant for binding target proteins, those that are cleaved by proteases, those that traverse cell membranes, etc. For most peptides of interest (5–20 amino acids), the ensembles contain thousands of low-energy conformations that can be further reduced through RMSD-based clustering. The second application, which we refer to as CMDboltzmann, is for estimating the binding affinity of a peptide sequence around a given backbone binding mode. It involves local peptide configurational sampling, subject to user provided constraints, to optimize a peptide binding mode with its protein target and the calculation of a predicted binding affinity from the resulting configurational ensemble. The chief difference between CMDpeptide and CMDboltzmann is that the latter involves placement of a peptide in a binding site, which requires inclusion of protein–peptide energetic interactions in any scoring function and explicit sampling of conformational and rigid-body coordinates. Additionally, while CMDpeptide assumes nothing about the structure of the peptide, CMDboltzmann uses different constraints to direct the peptide into the protein binding site. Fundamentally, both algorithms can be broken down into two loosely independent parts: sampling and scoring. Each of these is briefly described below, with particular emphasis on the differences between them.

In addition to the sampling-based methods, we describe several empirical and force field-based scoring functions for estimating protein–peptide binding affinities from single protein–peptide poses. These include our in-house empirical scoring function (CMDescore) and force field-based scoring function (CMDyscore). For comparative purposes, we also provide results obtained using the widely employed Xscore and Vina scoring functions along with protein–peptide surface area (SA) burial and interface packing (IP) calculations. The chief difference between these approaches and CMDboltzmann is that they are used to estimate the binding affinity from a single protein–peptide pose, whereas binding affinity estimates obtained using CMDboltzmann involve sampling thousands of softly constrained peptide backbone structures in the protein binding site. Because they only require a single pose the scoring functions are computationally much more efficient, making it possible to rapidly estimate binding affinities for thousands or even millions of peptide ligand sequences or binding poses in relatively short order.

The main goal of a typical peptide drug design project is to narrow the vast theoretical peptide sequence space down to a manageable number of viable candidate sequences that are predicted to bind a protein target of interest. A standard CMDInventus work-flow for accomplishing this would begin with combinatorial sequence scanning with a fixed peptide backbone, and rapid free energy scoring and ranking with CMDescore. Promising sequences would then be scored and prioritized using CMDyscore. The most promising peptide ligand sequences would then be scored for their protein binding affinities using the computationally expensive CMDboltzmann module. In addition, CMDboltzmann is useful for examining larger discrete changes to a peptide structure when the assumption of a fixed binding mode for the peptide ligand may no longer be valid and is somewhat relaxed (cyclization, large changes in side chains, changes in stereochemistry, etc.).

Full peptide conformational sampling with CMDpeptide. The purpose of CMDpeptide is the calculation of biologically relevant peptide conformational ensembles. All CMDpeptide conformational sampling is done in dihedral space, with fixed bond lengths and angles, using a multiple copy simulated annealing with minimization (MCSAM) algorithm [27,28,29]. Each run begins by initializing a stack of conformations by generating 100 conformations at random followed by energy minimization. During the run, the stack is allowed to grow to 200 conformations according to previously describe rules for maintaining the conformational stack [27]. For conformational sampling with CMDpeptide, only topological information, that is sequence information and any inter-residue bonds such as disulfide bonds, is used. Each run consists of 10,000 Monte Carlo w/Minimization (MCM) steps performed as described previously [27]. To ensure complete coverage, 500 independent runs are typically performed per peptide. In addition, Phi/Psi biasing is performed using pre-calculated Ramachandran plots. The plot for residue n within a given sequence (A_1_-A_2_-…-A_n_-…-A_N_-_1_-A_N_) is calculated by thoroughly sampling the tri-peptide Ac-A_n−1_-A_n_-A_n+1_-NH2. The reason for creating the plots in this fashion, rather than using plots derived from PDB structures, is the need for chemical and conformational generality. In particular, relying solely on PDB derived Ramachandran plots would effectively limit one to using the standard 20 amino acids. This would seriously limit the development and use of CMDInventus as a general computational framework for enabling peptide-based drug design using such things as non-natural side chains, D-amino acids, β-amino acids, α-di-substituted amino acids, and so forth.

Estimating binding affinities from conformational ensembles with CMDboltzmann. The primary goal of a CMDboltzmann calculation is to estimate the binding affinity of a protein–peptide binding mode where the peptide ligand is treated as semi-flexible. The binding mode for the peptide backbone would ideally derive from a co-crystal structure but can be derived from a docked or theoretically calculated binding mode. Importantly, CMDboltzmann can be used to calculate binding affinities as peptide sequences are mutated around a given binding mode. Thus, for CMDboltzmann calculations the simulation begins with a peptide backbone positioned in a binding site. While it is desirable for the peptide backbone to qualitatively maintain its binding configuration, significant changes in a side chain might require some movement of the peptide backbone. Hence, the backbone is permitted a limited range of softly constrained motions. To accomplish this, the same MCSAM algorithm described above is used with the following differences. The first and largest difference is the presence of the protein which greatly affects the scoring and subsequent ranking of each peptide configuration; the scoring of the protein–peptide interactions is described below in the sampling energy function section. For the calculations described here, the protein is held rigid, though in many cases we allow side-chain flexibility for residues of the protein in the binding site. The second difference is the presence of a constraint, described below in the sampling energy function section, that is applied to prevent the peptide backbone from deviating too greatly from its starting position. Typically, the backbone is constrained so that its atoms can move 1.0 Å without penalty. If a backbone atom moves more than 1.0 Å a harmonic penalty is applied. The third and final differences are details in the runs, including the number of steps per run (2000) and the total number of runs (100). Fewer steps and runs are needed because the conformational space being searched is far smaller due to the backbone constraints and the presence of the protein.

Force field used in calculations involving sampling. The Amber99sb force field provides all bonded interactions; because the sampling is done in dihedral space, this entails the use of only the Amber99sb dihedral energy terms. All nonbonded interactions within a peptide and between a protein and peptide are calculated as the sum of the Van der Waals interactions, the electrostatic interactions, and an empirical solvation term. The Van der Waals parameters are taken from Amber99sb [30]. For solvation, the EEF1 continuum model [31] is used, as it provides a reasonable balance between accuracy and speed. The electrostatic contribution to the system energy is calculated using a distance dependent dielectric function consistent with the recommendations of the EEF1 solvation model, i.e., the effective dielectric constant between two atoms is 4d where d is the distance between the two atoms. The atomic charges used for the electrostatic calculation are generated for each amino acid via the RESP [32,33] procedure applied to individual amidated and acylated amino acid building blocks using the quantum chemistry package GAMESS [34,35] at the B3LYP/6-31 G** level of theory. The final score calculated for a configuration of the peptide is simply the sum of these terms without any reweighting.

For the CMDboltzmann calculations, a constraint is applied to each heavy atom of the peptide ligand backbone (excluding the 2 terminal residues). The constraint is implemented as a harmonic well with a distance tolerance of 1.0 Å inside of which no penalty is applied. This allows the backbone to sample locally around the starting binding mode without deviating too much from the desired binding mode. Thus, for a CMDboltzmann calculation the MCSAM algorithm works to optimize the combination of backbone constraints + peptide internal energy + protein–peptide interaction energy. The final score for a given peptide sequence is the Boltzmann weighted, using the total energy, average of the protein–peptide interaction energy over the minimum energy pose from each of the 100 runs. In the present study, no attempt was made to estimate relative strain energies for different sequences, the assumption being that this is a minor contributor. In some cases, particularly with the linear α-helices, this may be poor assumption. In fact, recent work suggests that optimizing a sequence to stabilize a conformation is a viable option to improving potency [36,37,38,39,40,41,42,43].

Estimating binding affinities with CMDescore and CMDyscore from single pose structures. CMDescore [44,45,46,47] is a simple empirical scoring function for predicting binding affinities. It comes in a variety of flavors depending on the number of terms in the function. All flavors are premised on rigid-body binding and are parameterized for use on protein–protein and protein–peptide complexes composed of standard amino acids. For the present study, the flavor used has been described previously and is a linear combination of four regression weighted terms that quantify hydrophobic and charged group burial at a given protein–ligand interface and hydrogen bonding and salt bridge interactions across a given interface and is given by the following equation for binding affinity (BA) [47].

(1) $$BA=-1.94+0.16\cdot \Delta X_{H}-0.68\cdot \Delta X_{C}-0.52\cdot X_{HB}-0.41\cdot \Delta X_{SB}$$

The first term, *X_H_*, refers to the free energy change associated with hydrophobic group burial (carbon and sulfur atoms). The second term, *X_C_*, refers to the free energy change associated with charge group burial (nitrogen and oxygen atoms of charged D, E, K and R side chains). The third, *X_HB_*, and fourth terms, *X_SB_*, refer to the total number of conventional hydrogen bonds and net number of salt bridges calculated across a given protein–peptide interface.

CMDyscore [6,48,49] is a force-field-based scoring function based on the YASARA modeling and simulation package. As part of the scoring process, the mutated complexes were subjected to two rounds of minimization. During the first round all backbone atoms in the protein and peptide were fixed, and side chains in the protein that were less than four angstroms from the peptide and all side chains in the peptide were permitted to move. During the second round the entire protein is fixed and the entire peptide to allowed to move.

Four flavors of CMDyscore were used to calculate binding affinities: (1) simple, (2) unbound ligand minimized to apply a pseudo ‘strain’ penalty, (3) charged residues neutralized, and (4) implicit solvation model. The CMDyscore methods are implemented using YASARA [50]. Before calculating the scores, the complex was minimized using the NOVA force field [51]. The first method calculates the binding energy according to the formula:

(2) Energy(complex)−Energy(protein)−Energy(peptide)

The NOVA force field was used for the calculation of the energies of the individual protein, peptide and the protein–peptide complex. The complex was first minimized using the NOVA force field [51]. The NOVA force field was chosen because it is optimized for in vacuo minimizations, as necessitated for a fast scoring function. The energies for protein and peptide were calculated according to the rigid-body binding assumption, i.e., by extracting each in turn from the complex in the exact conformation that they assume in the complex.

The pseudo ‘strain’ penalty was originally implemented to handle the scoring of docking poses. One concern with docking is that in an attempt to maximize the docking protein interaction energy terms, the peptide ligand will be placed in an unrealistically high energy or strained conformation. This method introduces moderate peptide ligand flexibility into the binding energy calculation by minimizing the peptide after it is extracted from the complex:

(3) Energy(complex)−Energy(protein)−Energy(peptide, minimized)

If the binding conformation is not a low-energy conformation, the binding energy will be reduced, possibly significantly. This approach has been used in small molecule docking studies; Greenidge and coworkers, for example, included a similar term in when docking the PDBbind data set [52]. They did MM/GBSA calculations and found the best correlation (R^2^ = 0.63) with the strain energy term included and explicit waters excluded.

The latter two flavors represent alternate methods of damping the charge effects in the absence of explicit solvent. We have observed that peptides with charged residues are particularly hard to score. Even with the NOVA force field, peptides with charged side chains can appear as outliers. One way this problem was addressed was by neutralizing all the charged groups in the protein and peptide. The other way that this problem was addressed was to use a PBSA implicit solvent model. The PBSA model adds two terms to the energy: the polar and non-polar contributions to the solvation free energies. The former is determined by solving the Poisson-Boltzmann equation [53]. The latter is estimated from the solvent accessible surface area (SASA). A scaling factor of 0.65 was used for converting the surface area to free energy. The scaling factor comes from the YASARA manual. It was decided not to optimize the parameter based on the current data set, as it was felt that the initial results were reasonable and optimizing the value with a small data set would introduce undue bias.

In addition to CMDescore and CMDyscore, the docking score used by VINA [54], the Xscore [55] scoring function, and very simple scoring functions based solely on packing interface (PI) potential and buried surface area (SA) were also used. All were used according to their default settings and parameters.

Structure preparation for CMDescore and CMDyscore: In general, MDM2 and MDMX X-ray structures bound to reference peptides were prepared for subsequent calculations using standard cleaning and optimization procedures. This was followed by mutation or mutation and minimization of a reference peptide according to the relevant SAR data set. This, in turn, was followed by free energy scoring of all reference and mutated MDM2/MDMX targeting peptides.

Both the Li-p53 and Li-PMI datasets are alanine scanning datasets. Crystal structures for the Li-PMI data set are available for MDM2 and MDMX (3eqs and 3eqy). There are also crystal structures for the Li-p53 data set (4hfz and 1ycr for MDM2 and 3dab for MDMX). These crystal structures were used after applying a standard clean up routine (remove waters and counter ions, remove all but one of each chain, and add hydrogens).

For the Liu D-α-helical PMI-α alanine scanning peptide data set there are two relevant MDM2 crystal structures (3iwy and 3lnj). 3lnj was selected as the starting point. Because Asn2 has a seriously distorted geometry, it was removed and rebuilt. Because it was absent from the structure, Thr1 was built with standard α-helical Phi/Psi angles and an optimized side chain. There is no MDMX structural data for the Liu D-α-helical peptides.

There are crystal structures of stapled peptides bound to MDM2 and MDMX (3v3b and 4n5t). These were used as the starting points for mutation of the bound reference peptides to the sequence used in the Guerlavais ATSP-3900 stapled L-α-helix data set.

The cyclic peptides were all built from a single reference structure (2axi). A similar procedure to that used for the single residue mutations for the preceding data sets was used with the modification that mutations were done sequentially, the result of the previous single residue mutation serving as the input for the next mutation. The process was continued until the desired sequence was produced. As a result, the binding site was more fully minimized for the cyclic peptides than for the other data sets.

Several scoring functions were used to calculate binding affinities from the X-ray poses produced using the above-described procedures. In particular, two in house scoring functions were used:

To apply these scoring functions to the various MDM2 and MDMX data sets, co-crystal structures of reference peptides bound to MDM2 or MDMX binding sites were used as starting points. Peptide ligand residues were then either (1) mutated or (2) mutated and refined through a constrained minimization procedure according to the above-described data sets. Finally, all mutated structures were scored using all available single-point affinity estimation methods. More theoretical and computational details are provided below in the Results and discussion section.

Mdm2 Competitive Fluorescence Anisotropy Assay and K_d_ Determination Purified MDM2 (1-125) protein was titrated against 50 nM carboxyfluorescein (FAM)-labeled 12/1 peptide [56] (FAM-RFMDYWEGL-NH_2_). Dissociation constants for titration of MDM2 against FAM-labeled 12/1 peptide were determined by fitting the experimental data to a 1:1 binding model equation shown below: [57,58].

(4) $$r=r_{0}+\left(r_{b}-r_{0}\right)\left(\frac{\left(K_{d}+{\left[L\right]}_{t}+{\left[P\right]}_{t}\right)-\sqrt{{\left(K_{d}+{\left[L\right]}_{t}+{\left[P\right]}_{t}\right)}^{2}-4{\left[L\right]}_{t}{\left[P\right]}_{t}}}{2{\left[L\right]}_{t}}\right)$$

[*P*] is the protein concentration (MDM2), [*L*] is the labeled peptide concentration, *r* is the anisotropy measured, *r_o_* is the anisotropy of the free peptide, *r_b_* is the anisotropy of the MDM2–FAM-labeled peptide complex, *K_d_* is the dissociation constant, [*L*]*_t_* is the total FAM-labeled peptide concentration, and [*P*]*_t_* is the total MDM2 concentration. The determined apparent *K_d_* value of FAM-labeled 12/1 peptide (13.0 nM) was used to determine the apparent *K_d_* values of the respective competing ligands in subsequent competition assays.

Apparent *K_d_* values were determined for a variety of molecules via competitive fluorescence anisotropy experiments. Titrations were carried out with the concentration of MDM2 held constant at 250 nM and the labeled peptide at 50 nM. The competing molecules were then titrated against the complex of the FAM-labeled peptide and protein. Apparent *K_d_* values were determined by fitting the experimental data to the equations shown below: [58,59].

(5) $$\begin{array}{l} r=r_{0}+\left(r_{b}-r_{0}\right)\left(\frac{2\sqrt{\left(d^{2}-3e\right)}\cos\left(\frac{\theta }{3}\right)-9}{3K_{d1}+2\sqrt{\left(d^{2}-3e\right)}\cos\left(\frac{\theta }{3}\right)-d}\right)\\ d=K_{d1}+K_{d2}+{\left[L\right]}_{st}+{\left[L\right]}_{t}-{\left[P\right]}_{t}\\ e=\left({\left[L\right]}_{t}-{\left[P\right]}_{t}\right)K_{d1}+\left({\left[L\right]}_{st}-{\left[P\right]}_{t}\right)K_{d2}+K_{d1}K_{d2}\\ f=-K_{d1}K_{d2}{\left[P\right]}_{t}\\ \theta =\cos^{-1}\left(\frac{-2d^{3}+9de-27f}{2\sqrt{{\left(d^{2}-3e\right)}^{3}}}\right) \end{array}$$

[*L*]*_st_* and [*L*]*_t_* denote labeled ligand and total unlabeled ligand input concentrations, respectively. *K_d2_* is the dissociation constant of the interaction between the unlabeled ligand and the protein. In all competitive types of experiments, it is assumed that [*P*]*_t_* > [*L*]*_st_*, otherwise considerable amounts of free labeled ligand would always be present and would interfere with measurements. *K_d_*_1_ is the apparent *K_d_* for the labeled peptide used in the respective experiment, which has been experimentally determined as described in the previous paragraph. The FAM-labeled peptide was dissolved in dimethyl sulfoxide (DMSO) at 1 mM and diluted into experimental buffer. Readings were carried out with an Envision Multilabel Reader (PerkinElmer). Experiments were carried out in PBS (2.7 mM KCl, 137 mM NaCl, 10 mM Na_2_HPO_4_ and 2 mM KH_2_PO_4_ (pH 7.4)) and 0.1% Tween 20 buffer. All titrations were carried out in triplicate. Curve-fitting was carried out using Prism 4.0 (GraphPad, San Diego, CA, USA).

To validate the fitting of a 1:1 binding model we carefully determined that the anisotropy value at the beginning of the direct titrations between MDM2 and the FAM-labeled peptide did not differ significantly from the anisotropy value observed for the free fluorescently labeled peptide. Negative control titrations of the ligands under investigation were also carried out with the fluorescently labeled peptide (in the absence of MDM2) to ensure no interactions were occurring between the ligands and FAM-labeled peptide. In addition, we ensured that the final baseline in the competitive titrations did not fall below the anisotropy value for the free FAM-labeled peptide, which would otherwise indicate an unintended interaction between the ligand and the FAM-labeled peptide to be displaced from the MDM2 binding site.

## 4. Conclusions

CMDInventus is a modular computational package for performing peptide drug modeling calculations. The modules CMDpeptide and CMDboltzmann involve the explicit sampling of peptide conformational or configurational space and can be used to model and predict peptide conformations and protein–peptide binding affinities, respectively. CMDescore and CMDyscore are empirical and force field-based scoring functions, respectively, that can be used to rapidly predict protein–peptide binding affinities from single complex structures. All six methods were used to retrospectively reproduce diverse MDM2/MDMX-peptide data sets with an encouraging degree of success. CMDescore, CMDyscore and CMDboltzmann were used to prospectively and accurately predict the experimentally measured binding affinity results for an Ala-scan of the pharmaceutically relevant stapled peptide ATSP-7041. Remarkably, CMDboltzmann was used to successfully and accurately predict the results of a novel D-scan of ATSP-7041. All results were obtained without any re-fitting or re-parameterization. Collectively, our results suggest that CMDInventus is useful for retrospectively modeling and prospectively predicting the conformational and binding behavior for diverse and pharmaceutically relevant linear and macrocyclic α-helical peptides and that CMDInventus can serve as computational platform for enabling novel peptide drug design and discovery.

## Figures and Tables

**Table 1:** Datasets used as starting points for binding affinity scoring calculations.

Article	Peptide Ligand	MDM2	MDMX
Li	PMI	3eqs	3eqy
Li	p53	4hfz	3dab
Liu	D-α-helix	3lnj	3lnj/3eqy *
Sawyer	Stapled α-helix	3v3b	4n5t
Fasan	N–C cyclic peptide	2axi	

* 3lnj peptide docked in 3eqy.

**Table 2:** CMDpeptide results: Here, we summarize the full results of the CMDpeptide conformational analysis calculations. Each of these calculations begins with only sequence and inter-residue bonding information, i.e., only topological information. The “Number of AA” column lists the number of amino acids (not including the capping groups) and in parentheses lists the number of non-standard amino acids. The “Min RMSD” column gives the minimum RMSD conformation (Å) relative to the corresponding X-ray conformation of all conformations within 15 kcal/mol of the lowest energy conformation. Finally, the results from clustering with a radius of 1.0 Å are shown, including the total number of clusters (Size) and the lowest RMSD conformations within the top 25, 100 and 500 ranked clusters. All RMSDs are calculated over backbone atoms plus the C_β_ atoms. Average values are provided (the average value for cluster size has been rounded up to a whole number).

PDB	Description	Number of AA	Min RMSD (Å)	Cluster 1.0 Å			
				Size	25 (RMSD)	100 (RMSD)	500 (RMSD)
**1ycr**	L-α-helix (p53 17-29)	13 (0)	1.29	9547	2.9	2.2	2.1
**2axi**	N–C cyclic β-hairpin	10 (1)	0.77	744	1.4	1.3	1.3
**2gv2**	L-α-helix	8 (4)	1.17	3595	1.9	1.4	1.4
**3eqs**	L-α-helix	11 (0)	0.75	11,112	1.7	1.7	1.4
**3g03**	L-α-helix	11 (0)	0.94	9175	2.5	2.1	1.4
**3iux**	Stingin–2 disulfide bonds	18 (0)	1.34	4721	2.3	2.2	1.7
**3iwy**	D-α-helix	12 (12)	0.95	8074	2.1	1.8	1.5
**3jzo**	L-α-helix	12 (0)	1.02	7743	2.6	2.2	1.3
**3jzs**	L-α-helix	12 (0)	0.92	5368	2.6	2.6	1.5
**3lnj**	D-α-helix	11 (11)	0.88	3091	2.8	1.5	1.0
**3tpx**	D-α-helix	11 (11)	0.88	4784	2.4	1.7	1.1
**3v3b.**	Stapled α-helix	13 (2)	1.40	3674	2.5	2.3	1.8
**4n5t**	Stapled α-helix	14 (3)	1.44	4310	2.6	2.0	1.5
**4ud7**	Stapled α-helix	14 (2)	1.32	6484	3.2	2.5	1.5
**4umn**	Stapled α-helix	11 (2)	1.54	3201	2.2	1.9	1.7
**5afg**	Stapled α-helix	12 (2)	1.36	4354	2.4	1.5	1.5
		Average	1.12	5624	2.38	2.16	1.48

**Table:** **A.** Mutation + scoring (exluding inactives).

Method	R^2^ MDM2					R^2^ MDMX			Statistics	
	Li PMI	Li p53	Liu D-α	Guerlavais Stpl-α	Fasan Stpl-α	Li PMI	Li p53	Guerlavais Stpl-α	Avg	Stdev
CMDescore	0.831	0.031	**0.933**	0.556	0.420 ^1^	0.799	0.245	0.829	0.581	0.325
CMDyscore (minimize)	0.775	0.033	0.445	0.475	0.683	0.906	*0.013*	0.823	0.519	0.345
CMDyscore	0.837	0.027	0.463	0.507	**0.705**	0.908	*0.007*	0.837	0.536	0.357
CMDyscore (uncharged)	0.920	0.374	0.574	0.484	0.624	0.884	0.005	0.831	0.587	0.306
CMDyscore (solvation)	**0.930**	**0.485**	0.775	**0.609**	0.518	**0.958**	**0.575**	0.884	**0.717**	**0.193**
Xscore	0.890	0.290	0.726	0.540	0.442	0.850	0.434	0.816	0.624	0.226
VINA	0.912	0.300	0.682	0.493	0.534	0.893	0.233	0.833	0.610	0.263
Packing	0.870	0.330	0.677	0.551	0.418 ^1^	0.804	0.566	**0.921**	0.642	0.213
Buried SA	0.795	0.001	0.537	0.289	0.338	0.747	0.022	0.548	0.410	0.301

Bold means the largest in each column; Italics mean an inverse correlation; best prediction in bold; ^1^ CMDescore and packing interface score are not parameterized for non-canonical amino acids and so these were not included in the correlation; “Stpl-α” stands for stapled alpha helical peptide and “D-α” stands for D alpha helical peptide.

**Table:** **B.** Mutation + scoring (including inactives).

Method	R^2^ MDM2					R^2^ MDMX			Statistics	
	Li PMI	Li p53	Liu D-α	Guerlavais Stpl-α	Fasan Stpl-α	Li PMI	Li p53	Guerlavais Stpl-α	Avg	Stdev
CMDescore	0.831	0.466	**0.933**	0.556	0.420 ^1^	0.799	0.692	0.829	0.690	0.189
CMDyscore (minimize)	0.775	0.334	0.445	0.475	0.683	0.906	0.317	0.823	0.595	0.230
CMDyscore	0.837	0.332	0.463	0.507	**0.705**	0.908	0.328	0.837	0.615	0.236
CMDyscore (uncharged)	0.920	0.815	0.574	0.484	0.624	0.884	0.717	0.831	0.731	**0.157**
CMDyscore (solvation)	**0.930**	**0.891**	0.775	**0.609**	0.518	**0.958**	**0.909**	0.884	**0.809**	0.163
Xscore	0.890	0.835	0.726	0.540	0.442	0.850	0.863	0.816	0.745	0.166
VINA	0.912	0.835	0.682	0.493	0.534	0.893	0.801	0.833	0.748	0.161
Packing	0.870	0.859	0.677	0.551	0.418 ^1^	0.804	0.883	**0.921**	0.748	0.182
Buried SA	0.795	0.523	0.537	0.289	0.338	0.747	0.545	0.548	0.540	0.174

Bold means the largest in each column; Italics mean an inverse correlation; best prediction in bold; ^1^ CMDescore and packing interface score are not parameterized for non-canonical amino acids and so these were not included in the correlation; “Stpl-α” stands for stapled alpha helical peptide and “D-α” stands for D alpha helical peptide.

**Table 4:** Prospective Testing Data.

Residue	Experimental MDM2		Experimental MDMX		Calculated MDM2 Calculated Binding *		
	Kd (nM)	ΔΔG (kcal)	Kd (nM)	ΔΔG (kcal)	CMDescore ΔΔG (kcal)	CMDyscore ΔΔG (kcal)	CMDboltzmann ΔΔG
**WT**	18.6		12.5				
**L1A**	19.3	0.03	9.6	−0.16	0.2	−0.30	1.2
**T2A**	155.3	1.73			−0.2	−0.91	1.3
**F3A**	9585.9	5.09	4873	3.53	1.8	27.28	7.8
**E5A**	29.8	0.38	45.6	0.75	0.0	1.81	−1.4
**Y6A**	51.5	0.83	130.3	1.37	0.7	9.42	0.4
**W7A**	5145.9	4.58	11161	4.02	3.8	36.41	11.6
**Q8A**	32.6	0.46	57.1	0.88	0.3	1.71	−1.0
**L9A**	15.8	−0.13	13.4	0.00	0.2	0.42	−0.7
**Cba10A**	413.7	2.53	94.7	1.18	0.8	10.74	7.1
**S12A**	20.4	0.08	43.9	0.73	0.2	−2.29	1.3
**L1dL**	27.5	0.23	21.8	0.31	0.0	−1.90	−1.3
**T2dT**	217.8	1.46			0.3	−2.24	−2.6
**F3dF ^b^**	6385.1	3.47	7397	3.77	−0.2	−4.04	5.7
**E5dE**	36.8	0.41			−0.5	−2.44	1.5
**Y6dY**	365.1	1.77	921.2	2.53	0.7	5.91	0.7
**W7dW ^b^**	32.9	0.34	63.4	0.94	0.2	−1.73	2.2
**Q8dQ**	26.5	0.20	36.6	0.62	0.2	−0.93	2.3
**L9dL**	32.2	0.32	38.8	0.65	0.0	−1.08	−2.5
**Cba10dCba**	3809.5	3.17	805.1	2.45	1.3	8.21	3.5
**S12dS**	10.7	−0.31	36.9	0.62	−0.5	−2.45	0.1
**A13dA**	17.2	−0.05	34.9	0.59	0.3	−0.69	0.0
**A14dA**	18.4	−0.02	4.9	−0.57	−0.3	4.31	−1.9

* The MDM2 and MDMX data are very similar; MDMX data is excluded to avoid redundancy and minimize clutter. ^b^ All three methods were used to make the successful but surprising prediction that the chiral amino acid substitution W7 → dW7 would be weakly destabilizing; only CMDboltzmann was used to successfully predict the strongly destabilizing effect of F3 → dF7. This nicely illustrates the important role a more computationally expensive algorithm like CMDboltzmann can play in prospective peptide ligand candidate design and optimization.
